# Effects of Taiwan’s COVID-19 alert levels on the physical activity behaviors and psychological distress of community-dwelling older adults

**DOI:** 10.1186/s12877-023-04035-5

**Published:** 2023-05-19

**Authors:** Wang-Sheng Lin, Chih-Chun Tsai, Po-Jung Pan

**Affiliations:** 1 Department of Physical Medicine & Rehabilitation, Taipei Veterans General Hospital, Yuan-Shan/Su-Ao Branch, Yilan, Taiwan; 2 School of Medicine, National Yang Ming Chiao Tung University, Taipei, Taiwan; 3 Department of Mathematics, Tamkang University, Taipei, Taiwan; 4 Department of Physical Medicine & Rehabilitation and Center of Community Medicine, National Yang Ming Chiao Tung University Hospital, Yilan, Taiwan

**Keywords:** Sars-CoV-2, Exercise, Mental health, Lifestyle, Aging, Lockdown

## Abstract

**Background:**

The Taiwanese government implemented stringent preventative health measures to curb the spread of COVID-19. However, these measures negatively affected the physical activity behaviors and psychological distress of individuals. In this study, we investigated the effects of Taiwan’s COVID-19 alert–based restrictions on the physical activity behaviors and psychological distress of community-dwelling older adults.

**Methods:**

In this longitudinal study, 500 community-dwelling older adults were randomly sampled from a health promotion center in Taiwan. Telephone interviews were conducted between May 11, 2021, and August 17, 2021, which coincided with the Level 3 alert period when group physical activities were prohibited. Telephone interviews were again conducted between June 20, 2022, and July 4, 2022, after the alert level was reduced to Level 2 but group physical activities were prohibited period. Through the telephone interviews, data regarding the participants’ physical activity behaviors (type and amount) and 5-item Brief Symptom Rating Scale (BSRS-5) scores were collected. Moreover, data regarding physical activity behaviors were collected from the records of our previous health promotion programs, which were conducted before the national alert period. The obtained data were analyzed.

**Results:**

The alert levels influenced physical activity behaviors. Because of strict regulations, physical activity amount decreased during the Level 3 alert period and did not recover rapidly during the Level 2 alert period. Instead of engaging in group exercises (e.g., calisthenics and qigong), the older adults chose to exercise alone (e.g., strolling, brisk walking, and biking). Our findings indicate that the COVID-19 alert level has a significant influence on the amount of physical activity for participants (*p* < 0.05, partial η2 = 0.256), with pairwise comparisons showing that the physical activity amount decreased significantly across the three time periods (*p* < 0.05). The psychological distress of the participants did not appear to change during the regulation period. Although the participants' overall BSRS-5 score was slightly lower during the Level 2 alert period compared to the Level 3 alert period, the difference was not statistically significant (*p* = 0.264, Cohen's d = 0.08) based on a paired t-test. However, the levels of anxiety (*p* = 0.003, Cohen's d = 0.23) and inferiority (*p* = 0.034, Cohen's d = 0.159) were considerably higher during the Level 2 alert period than during the Level 3 alert period.

**Conclusions:**

Our findings indicate that Taiwan’s COVID-19 alert levels influenced the physical activity behaviors and psychological distress of community-dwelling older adults. Time is required for older adults to regain their prior status after their physical activity behaviors and psychological distress were affected by national regulations.

## Introduction

The coronavirus disease 2019 (COVID-19) pandemic has caused national governments to implement stringent lockdown measures. Taiwan’s Central Epidemic Command Center implemented a four-level COVID-19 alert system alongside several prevention measures to manage the unpredictable and rapid surge in case numbers that began on May 19, 2021. Subsequently, a nationwide Level 3 epidemic alert was declared. During this period, Taiwan’s government implemented mandatory facial masking; required registration for entry into all indoor facilities; cancelled all outdoor and indoor gatherings involving more than 10 and 5 people, respectively; closed schools from kindergarten through college; and promoted nonmandatory work-from-home work arrangements. Although COVID-19 can affect people of all ages, older adults are more susceptible to unfavorable consequences and death due to COVID-19 [1]. The accumulation of numerous harmful alterations in cells and tissues, which ultimately leads to the onset of chronic illness, tends to increase exponentially during the fifth decade of life [2]. Older adults, particularly those with comorbidities, are highly susceptible to fatal infections and other major health problems because of their reduced immune function [3, 4]. The rates if severe infection in individuals aged 50–64, 65–79, and ≥ 80 years were 19.8%, 43.2%, and 81.3%, respectively, and the corresponding mortality rates were 1.2%, 4.5%, and 18.8% [4]. Various governments introduced containment measures, such as isolating individuals with suspected COVID-19, to curb the spread of COVID-19 and prevent the collapse of health-care systems [5]. To prevent infections, older adults in Taiwan were specifically encouraged to stay at home whenever possible. The ensuing quarantine measures encouraged a sedentary lifestyle [6] and considerably reduced physical activity behaviors [7–9], resulting in increased psychological disorders (e.g., depression, anxiety, and posttraumatic symptoms) [10, 11] and poor sleep quality [9, 12–14].

Increasing evidence suggests that COVID-19 affects people’s general well-being, including their mood, sleep, and physical activity. An international online survey on COVID-19 involving 5056 participants revealed that home confinement exerted considerable negative effects on sleep habits and physical activity levels [15]. Home confinement reduced the frequency of a good sleep from 61 to 48%. During COVID-19-related home confinement, the total time spent on physical activity decreased substantially and the daily sitting time increased by 2 h. An online survey was conducted among Iranian individuals to explored how COVID-19-related public health regulations affected people’s physical activity, anxiety, well-being, and sleep quality. Compared with the data of sedentary participants, active participants exhibited improved well-being, enhanced sleep quality, and reduced anxiety; this indicated the positive effects of physical activity on well-being, anxiety, and sleep quality during the implementation of COVID-19 restrictions, which appeared to reduce physical activity behaviors [16]. The aforementioned finding further clarifies the effect of COVID-19 on psychophysical health conditions. Instead of focusing on specific aspects of psychophysical health, health-care professionals should assess it comprehensively.

Physical and mental health are crucial aspects of healthy aging. When people age, they are more likely to develop chronic health conditions such as arthritis, heart disease, and diabetes [17]. However, regular exercise and a balanced diet can help people maintain their physical health and prevent the onset of the aforementioned conditions [18]. For older adults, the recommended dose of physical activity is ≥ 150 or ≥ 75 min of moderate- or vigorous-intensity aerobic physical activity, respectively, per week. In addition, they should perform muscle-strengthening activities ≥ 2 days a week. Physical activities suitable for older adults include walking, cycling, swimming, yoga, and tai chi [19–21]. Before the COVID-19 pandemic, numerous older adults in Taiwan participated in group activities, such as dance classes, group fitness classes, and sports leagues. However, the pandemic has resulted in the cancellation of various activities or the modification of group activities to accommodate social distancing measures. A study involving 517 older adults [22] revealed that the COVID-19 lockdown substantially reduced the participants’ mental health, sleep quality, and total physical activity levels (in terms of energy expenditure); it also indicated that changes in Pittsburgh Sleep Quality Index (PSQI) scores and the total energy used during physical activities were key predictors of the decline in mental health from the prelockdown to lockdown periods. Another study involving 13,881 adult participants from 18 regions explored whether the pandemic altered the participants’ preferences for specific types of physical exercises [23]; during the pandemic, many people continued to exercise; several people opted to run instead of engaging in competitive sports (e.g., tennis and soccer). Despite the social distancing–related limitations, the participants generally continued to perform their preferred exercises [23]. However, national governments have implemented their own lockdown measures, leading to diverse developments worldwide. Therefore, how physical activity behaviors in Taiwan varied over time because of the changes in lockdown levels is a topic that warrants further exploration.

The COVID-19 pandemic and the subsequent implementation of home confinement measures substantially affected the mental health of individuals [24–26]. The sudden and unanticipated changes in daily life and the fears of illness and financial insecurity led to increased stress and anxiety in many people. The long duration of isolation and lack of social interaction also exacerbated feelings of loneliness and depression [27]. Furthermore, the blurring of the boundaries between work and home life and the burden of caring for family members increased the levels of burnout and fatigue. This effect was particularly pronounced in high-risk groups, such as front-line workers [25, 26] and individuals with preexisting mental health conditions [25]. Overall, the COVID-19 pandemic highlighted the necessity of addressing and mitigating the negative effects of home confinement on mental health. Thus, the psychological effects of COVID-19 home confinement in Taiwan at various alert levels should be comprehensively explored before future public health responses.

In Taiwan, the government downgraded the epidemic alert level to Level 2 on July 27, 2021. Under Level 2 epidemic prevention regulations, older adults could participate in physical activities, provided that they wore masks, frequently washed their hands, received the third dose of COVID vaccination, tested negative in their weekly rapid screening tests, and maintained a social distance with others (1.5 and 1 m for indoor and outdoor environments, respectively). Group physical activities were put on a hold for at least 1 week if someone in a community was infected with COVID-19. However, group physical activities were at one time not allowed between May 15, 2022, and June 30, 2022, because of COVID-19 outbreak. To the best of our knowledge, no longitudinal study has explored the effects of COVID-19 on the psychophysical health of older adults during various alert periods in Taiwan. Assessing if different isolation levels exert the same effect on health is essential to understanding the long-term implications of lockdown measures on the physical activity behaviors and psychological distress of community-dwelling older adults.

The COVID-19 pandemic considerably affected the lifestyles of people and might have had a substantial effect on psychophysical health conditions. Therefore, the present study was conducted to investigate the effects of Taiwan’s COVID-19 alert restrictions on the physical activity behaviors and psychological distress of community-dwelling older adults.

## Participants and methods

### Study design and participants

**Fig. 1:**
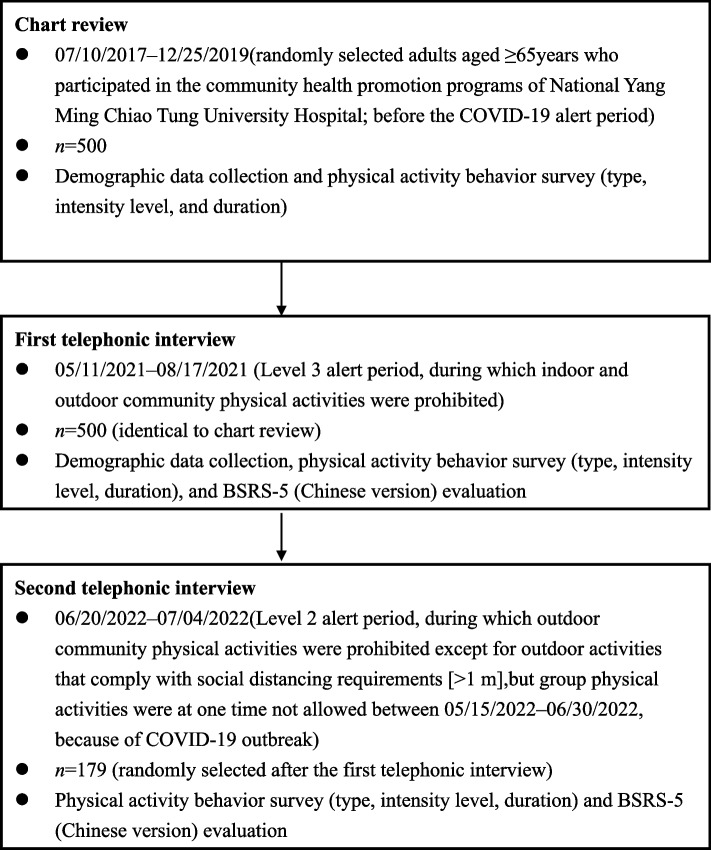
Flowchart of study protocol

Against the backdrop of the evolving SARS-CoV-2 pandemic and the slowing down of its spread, the present study was conducted as a retrospective chart review. In total, 500 older adults who had participated in community-based physical activities and visited the Center of Community Medicine at National Yang Ming Chiao Tung University Hospital to attend community health promotion programs [28, 29] were randomly selected. These individuals were interviewed through telephone by two fitness coaches and one trained assistant between May 11, 2021, and August 17, 2021, during which restrictions were imposed on group physical activities. After the alert level was downgraded to Level 2, telephone interviews were conducted again between June 20, 2022, and July 4, 2022, during which outdoor physical activities were still prohibited. Data regarding physical activity (type and amount) and 5-item Brief Symptom Rating Scale (BSRS-5) scores were collected through the telephonic interviews. In addition, data regarding physical activity behaviors (type and amount) were collected from the records of our previous health promotion program, which were conducted during the prealert period (before the implementation of COVID-19-related regulations). Figure 1 presents a flowchart showing the study protocol. Individuals were included in the present study if they were at least 65 years old and a resident of Yilan County, had no severe medical problems, and could participate in the health promotion program. Individuals were excluded if they had severe neurological impairments, had considerable cognitive impairments, had experienced recent traumatic events, had acute illnesses (e.g., unstable cardiac dysrhythmia, sepsis, and unstable vital signs), or were fully dependent on caregivers for daily activities.

All procedures in the present study were performed in accordance with the ethical principles established by the World Medical Association Declaration of Helsinki and National Yang Ming Chiao Tung University Hospital. The present study was reviewed and approved by the Human Subject Research Ethics Committee of National Yang Ming Chiao Tung University Hospital (approval number, 2020A012). Written informed consent for participation was obtained from all participants. To maintain the credibility of our manuscript, we would like to declare that there are no financial or personal relationships that could potentially affect the content of this paper. We confirm that we did not use artificial intelligence at any stage in writing this manuscript, and our research is based solely on our own statistical analysis and interpretation of data, with no conflicts of interest [30].

### Data collection

Eligible older adults were interviewed through telephone by the researchers. The study procedure involved demographic data collection; physical activity behavior survey, which collected data on the type and amount of physical activity performed (physical activity amount = intensity level of each physical activity type × duration of physical activity); and BSRS-5 (Chinese version) evaluation.

### Outcome measures

#### Demographics

During the interviews, skilled researchers collected data regarding the participants’ demographics (e.g., age, sex, residence, and physical activity characteristics).

#### Types of physical activities

Through a questionnaire, the participants were inquired about their involvement in physical activities and the type of physical activities they engaged in (depending on whether they answered “yes” or “no” to the first question). All activities that the participants listed in this survey as “their activities” were regarded as physical activities, and this approach was clearly communicated to them. A broad definition was applied to physical activities, which included calisthenics, jogging, brisk walking, and various other activities but excluded activities that people performed as part of their jobs. While completing the questionnaire, the participants could skip an item and provide a free-text response. For each activity type, the number of participants who engaged in it was recorded; using the sum of all the participant numbers as the denominator, the proportions of participants for all identified activity types were calculated.

#### Amount of physical activity

Through the telephonic interviews, data on the physical activities performed by each participant in the 1 week preceding their interview were collected. On the basis of the Taiwanese version of the International Physical Activity Questionnaire-Short Form [31, 32], physical activities were categorized by energy requirement into low-intensity (e.g., walking; 3.3 metabolic equivalents of task [METs]), moderate-intensity (4 METs), and high-intensity (8 METs) physically activities. The amount of physical activity performed was calculated by multiplying the intensity level of a given activity type by the duration of the activity. Amount of physical activity was expressed in terms of MET-min/week.

##### BSRS-5

For our telephonic interviews, we used the Chinese version of the 5-item BSRS-5, which measures psychological distress; the responses are rated on a Likert scale with end points ranging from 0 to 4) [33–35]. Five questions were posed to the participants to inquire whether they were feeling tense or keyed up (anxiety), feeling low (depression), feeling easily annoyed or irritated (hostility), feeling inferior to others (interpersonal hypersensitivity: inferiority), and having trouble falling asleep (insomnia).

The response to each question was scored on a Likert-type scale with end points ranging from 0 and 4 (0, *not at all*; 1, *a little*; 2, *moderately*; 3, *considerably*; 4, *extremely*). A BSRS-5 score of > 14 suggests severe psychological distress. The score ranges of 10–14 and 6–9 suggest moderate and low levels of psychological distress, respectively. In this study, the BSRS-5 scale exhibited acceptable consistency (Cronbach’s alpha = 0.67–0.75).

### Statistical analysis

The descriptive statistics used to describe the demographics of the participants comprised means, standard deviations, and percentiles. Repeated measures analysis of variance was performed to compare the results corresponding to the Levels 3 and 2 alert periods in terms of the amount of physical activity. Paired *t* tests were performed to compare the results corresponding to the Levels 3 and 2 alert periods, specifically the individual BSRS-5 scores for each question and the overall BSRS-5 score. Statistical analyses were performed using SPSS (version 22.0; IBM, Armonk, NY, USA). A *p* value of less than the significance level of 0.05 indicated statistical significance.

## Results

### Descriptive statistics

In the present study, 500 older adults aged ≥ 65 years (men, 104; mean age, 73.2 ± 7.3 years) were recruited to participate in telephonic interviews during the Level 3 alert period. All participants lived in Yilan County, which is less urbanized and less densely populated than many of Taiwan’s large cities. Because of the rapidly changing pandemic, the prohibition on group physical activities during the Level 2 period lasted for approximately 1.5 months, during which only 179 s-round telephonic interviews were completed. During the initial survey, 97.1% of all participants reported having a physical activity habit and 93.5% indicated that they engaged in physical activities > 3 times a week. In terms of intensity, 23.4% 76.5%, and 0.1% of all participants engaged in low-, moderate-, and high-intensity physical activities, respectively. During the final survey, which was conducted after the implementation of COVID-19 prevention measures, 81% of all participants indicated that they had a physical activity habit and 70.4% reported engaging in physical activities > 3 times a week. Most individuals engaged only in low-intensity (63.1%) and moderate-intensity (36.9%) activities. Table 1 summarizes the participants’ demographics.

**Table 1:** Participant demographics

	**Before alert**	**Level 3 COVID-19 alert**	**Level 2 COVID-19 alert**
Number	500	500	179
Male: Female (%)	20.8: 79.2	20.8: 79.2	24.0: 76.0
Age (years old)	73.2 ± 7.3	74.7 ± 7.1	75.9 ± 6.5
With physical activity habit(%)	97.1	83	81
> 3 /week physical activity (%)	93.5	77.6	70.4
Physical activity intensity (%) low: moderate: high^*^	23.4: 76.5: 0.1	54.2: 45.8: 0	63.1: 36.9: 0

^*^Low intensity, approximately 3.3 METs; moderate intensity, approximately 4 METs; high intensity, approximately 8 METs. MET, metabolic equivalent of task

### Longitudinal changes in the types of physical activities performed by Taiwan’s community-dwelling older adults

**Fig. 2:**
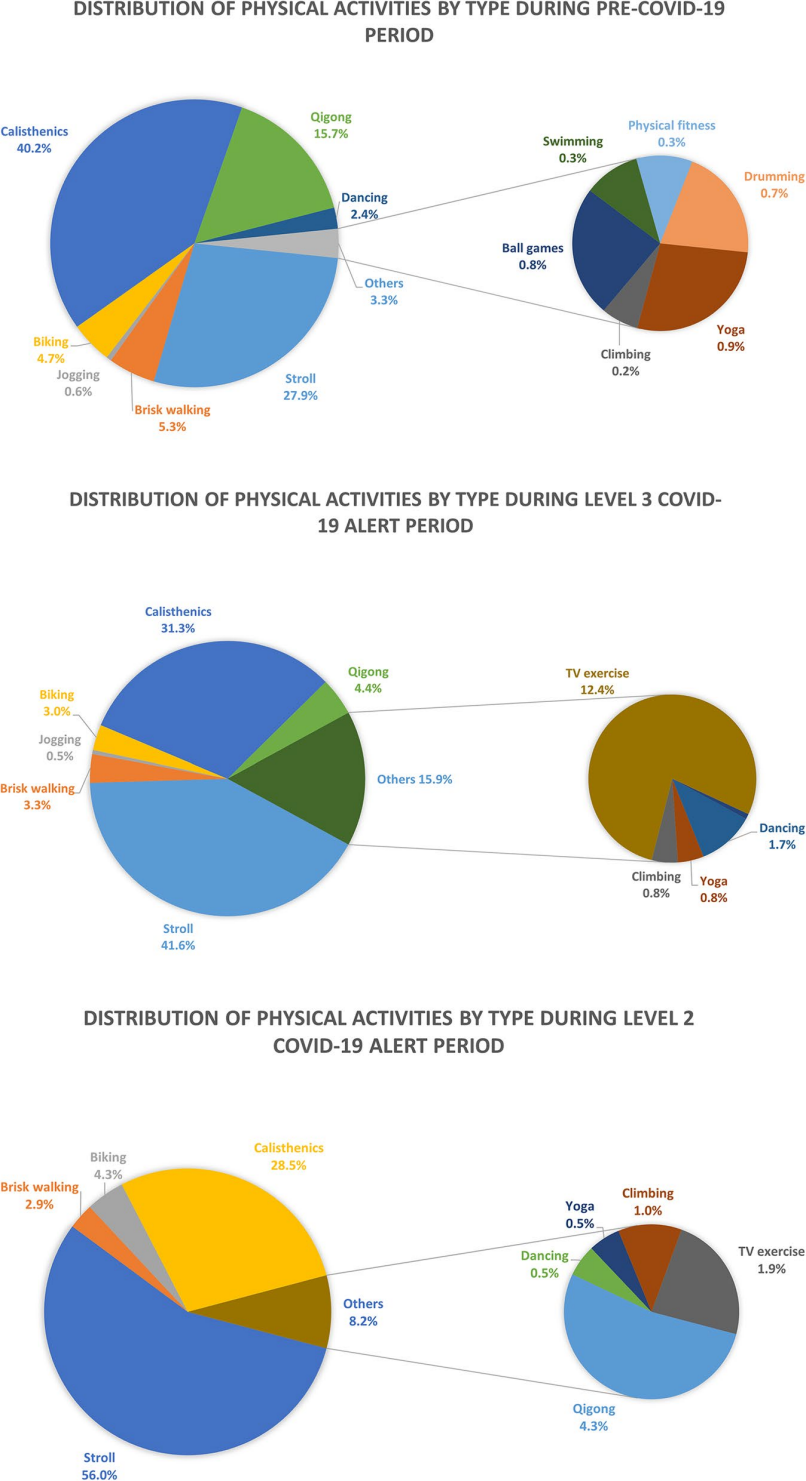
Distribution of different physical activities during the prealert, Level 3 alert, and Level 2 alert periods

Before the implementation of COVID-19-related regulations, calisthenics (40.2%), strolling (27.9%), and qigong (15.7%) were the most popular types of physical activities among the participants. During the Level 3 alert period, strolling (41.6%), calisthenics (31.3%), and other activities (15.9%) were the most popular physical activities among the participants. During the Level 2 alert period, the most popular activities among the participants were strolling (56.0%), calisthenics (28.5%), and others (8.2%). Figure 2 depicts the distribution of different physical activities during the prealert, Level 3 alert, and Level 2 alert periods.

### Longitudinal changes in the amount of physical activity performed by Taiwan’s community-dwelling older adults

**Fig. 3:**
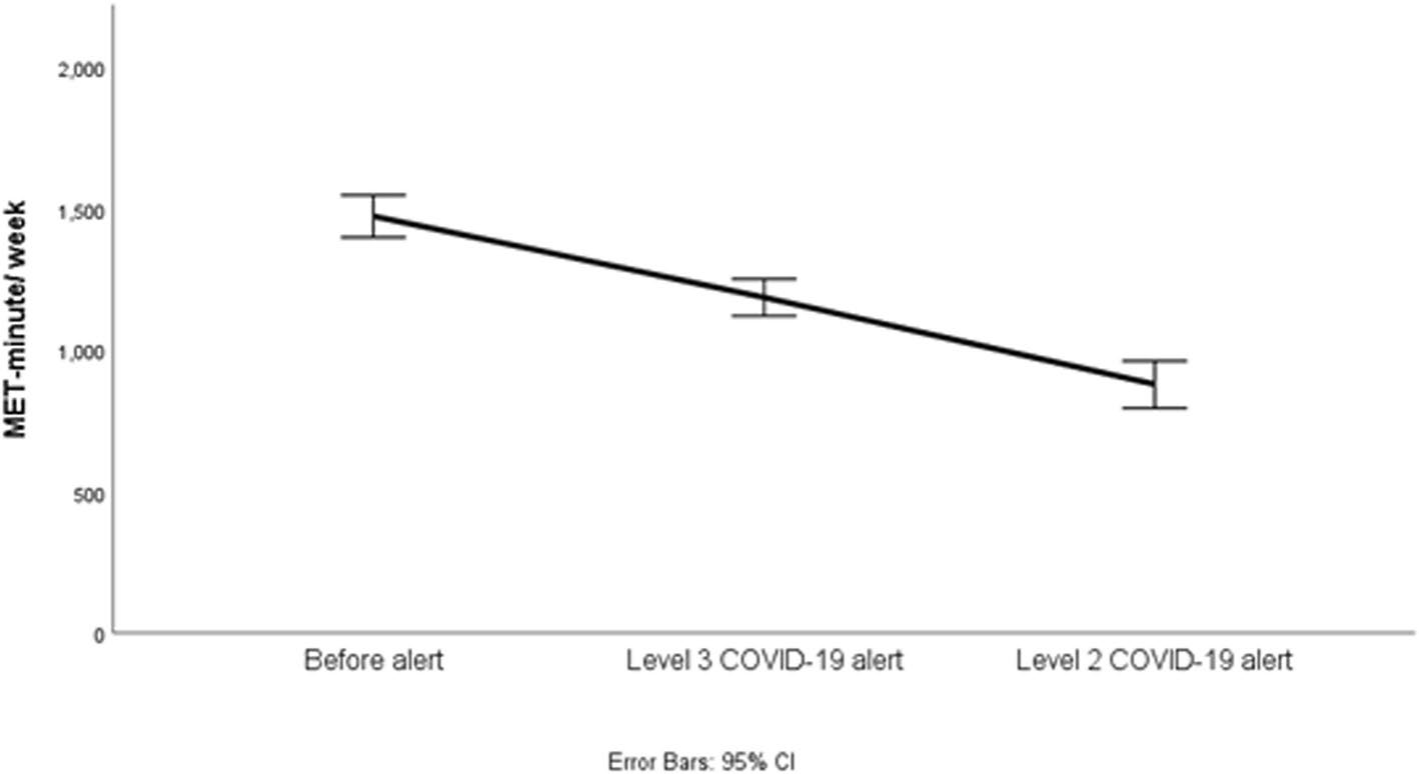
Changes in the amounts of physical activities at various alert levels

The amount of physical activity performed decreased during the Level 3 alert period compared with the amount during the prealert period. The amount decreased more during the Level 2 alert period than during the Level 3 alert period. The amount of physical activity did not increase until the lockdown was withdrawn. To analyze changes in physical activity amount over three periods, we employed repeated measures ANOVA. Violation of the sphericity assumption (*p* < 0.05) required adjustments to degrees of freedom. Our results indicated a significant effect of time on physical activity levels (partial η^2^ = 0.256), with pairwise comparisons showing significant differences between at least two of the periods and a decrease in physical activity overall. Figure 3 presents the amounts of physical activities performed during the prealert, Level 3 alert, and Level 2 alert periods.

### Longitudinal changes in the BSRS-5 scores of Taiwan’s community-dwelling older adults

**Fig. 4:**
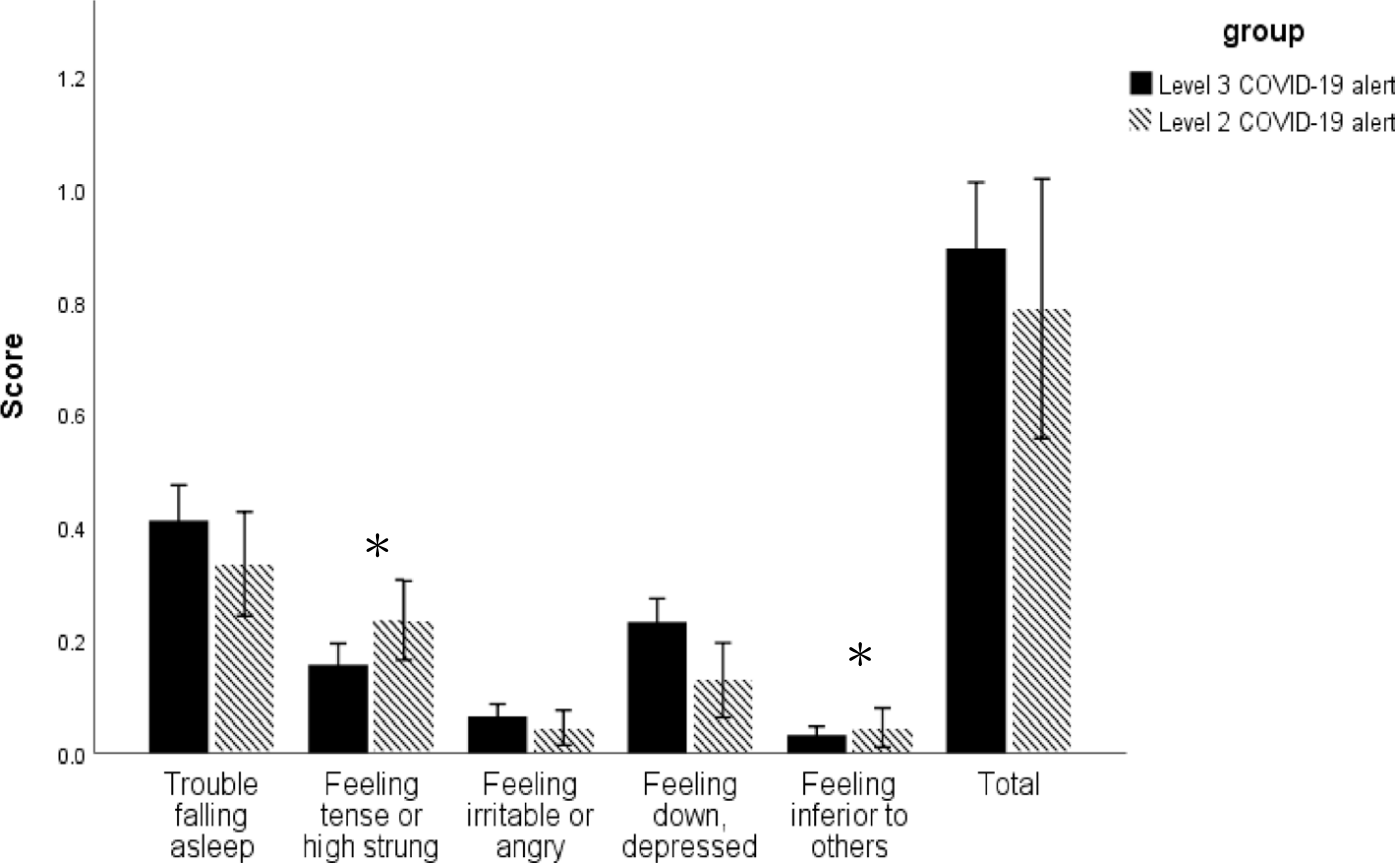
Changes in BSRS-5 scores from Level 3 to Level 2 alert periods

Our findings indicated that the participants’ mental health remained unchanged during the regulation period. This was indicated by the participants’ overall BSRS-5 scores and their scores for each item, which were < 6. Although the participants’ overall BSRS-5 score was lower during the Level 2 alert period than during the Level 3 alert period, the difference was nonsignificant (*p* = 0.264, Cohen's *d* = 0.08). The individual item scores indicated that the participants experienced considerably more anxiety (*p* = 0.003, Cohen's *d* = 0.23) and inferiority (*p* = 0.034, Cohen's *d* = 0.159) during the Level 2 alert period than during the Level 3 alert period. The BSRS-5 scores corresponding to the Levels 3 and 2 alert periods are presented in Fig. 4.

## Discussion

Promoting physical activity is a key health promotion strategy for community-dwelling older adults. To prevent and delay disability in older adults, a program based on the National Ten-Year Long-Term Care Plan 2.0 was launched in Taiwan in 2015. Community-dwelling older adults were encouraged to participate in various health promotion activities within their communities, such as exercise and dance programs at long-term community care centers. Through these programs, older adults developed a habit of performing exercises by themselves or participating in physical activity sessions at community centers. However, these programs were affected by the COVID-19 pandemic. In Taiwan, COVID-19 affected the physical activity behaviors of community-dwelling older adults prior to the implementation of the epidemic alert system, during the Level 3 alert period, and during the Level 2 alert period. We also evaluated the effects of COVID-19 on the psychophysical health of community-dwelling older adults during the Levels 3 and 2 alert periods. Our findings revealed that the most popular types of physical activity during the COVID-19 lockdown were strolling and calisthenics. The participants continued to engage in physical activities but switched from group activities to solitary activities. The participants’ physical activity levels decreased after stringent regulations were imposed during the Level 3 alert period and did not increase again even after the alert level was downgraded to Level 2. From the Level 3 to Level 2 alert periods, the participants exhibited improvements in overall BSRS-5 scores. However, they experienced more anxiety and inferiority during the Level 2 alert period than during the Level 3 alert period. Therefore, the COVID-19 epidermic alert in Taiwan negatively affected the participants’ assessments of their physical activity behaviors and psychological conditions.

Older adults must engage in ≥ 150 or ≥ 75 min of moderate- or high-intensity aerobic physical activity per week. Resistance training (e.g., lifting weights) should also be performed at least 2 days a week [19–21]. However, because of the epidemic, numerous events were cancelled or modified to comply with social distancing requirements. A COVID-19 worldwide study involving 5056 participants revealed that home confinement resulted in considerable negative changes in sleep patterns and physical activity levels [15]. During home confinement, the time spent by the participants on physical activities decreased significantly, whereas the daily sitting time increased by 2 h. Furthermore, a review study suggested that during the pandemic, school closure, lockdown, social isolation, and online learning increased the frequency of screen use [6], which promoted a sedentary lifestyle. Furthermore, an online survey of 3323 participants revealed a general decrease in physical activity because of the restrictions; among the participants who were determined to be physically inactive, 60.6% became less active and 5.1% became more active; moreover, among the participants who were determined to be physically active, 49.9% became less active and 22.8% became more active [16]. Our findings corroborate those of other studies indicating that physical activity levels decreased after the implementation of stringent COVID-19 regulations during the Level 3 and 2 alert periods. During pandemic situations, public health strategies must be implemented to enhance people’s awareness regarding the negative health effects of a sedentary lifestyle, which leads to noncommunicable illnesses. Solutions should limit the immobilization of older adults and promote the adoption of regular exercise habits among this population.

Walking, cycling, swimming, yoga, and tai chi are examples of recommended physical activities for older adults [19–21]. Many older adults in Taiwan participated in group physical activities (e.g., dancing courses, group fitness programs, and sports leagues) before the COVID-19 pandemic. However, after the Taiwanese government issued the national Level 3 epidemic alert, the highly stringent restrictions that followed negatively affected the healthy behaviors of individuals [36, 37]. Additionally, the reduced number of gyms and sports facilities and low social mobility limited physical activities [38]. Because of various precautionary measures, the types of physical activities that people engaged in changed depending on the alert level. Several similarities and differences can be noted between our findings and those of studies conducted in Western countries. In Taiwan, numerous community-dwelling older adults chose to exercise alone (e.g., strolling, brisk walking, and biking) instead of engaging in group activities (e.g., calisthenics or qigong). This is because solitary exercises can be performed outdoors, away from people, and with some basic tools and minimal experience. For these reasons, solitary physical activities were not significantly affected by COVID-19 restrictions and could be readily modified to comply with these restrictions. Furthermore, during the national alert period, solitary physical activities could be performed indoors without violating the restrictions on indoor gatherings (i.e., no more than five people). In contrast to another study, the present study revealed that people’s preferences for specific types of physical activities varied during the pandemic [23]. Many people could resume exercising; however many chose to exercise alone (e.g., running) rather than in groups (e.g., playing tennis and football) [23]. Our findings suggest that although Taiwan’s severe regulations prevented the spread of COVID-19, these regulations affected the physical activity preferences of community-dwelling older adults.

In retrospective cross-sectional study involving 1454 elite athletes, 29% of all participants reported maintaining their training intensity during the lockdown, whereas the other 71% reduced their training intensity. PSQI and insomnia severity index (ISI) scores were higher during the lockdown than during the prelockdown period; this was associated with the longer sleep onset latency and later preferred time of the day for the training of the participants. Participants who reduced their training intensity during the lockdown period had higher PSQI and ISI scores than did those who maintained their training intensity. Although the duration of lockdown exerted no effect on PSQI scores, athletes who were confined for longer periods had higher ISI scores than did those who were confined for only a month or less [14]. For older adults, starting slowly and gradually increasing the intensity and duration of physical activity is crucial; older adults should also consult their health-care providers before following a new exercise program [19–21]. In the present study, 23.4% of all participants engaged in low-intensity physical activities, with 76.5% engaging in moderate-intensity activities and 0.1% engaging in high-intensity activities. Our findings pertaining to the period after the implementation of COVID-19 prevention measures are consistent with those of other studies [9, 14]; specifically, most participants adjusted their physical activity behaviors (e.g., reducing training intensity and frequency) and fewer older individuals had physical activity habits. Health promotion programs should be designed considering these behavioral changes and methods for sustaining participation in physical activities. This is because maintaining exercise habit and healthy eating habits are advantageous for older adults; such habits may help them preserve their physical and mental health and reduce the risks of chronic illnesses [18].

In a relevant study, the effects of 4-month-long home confinement were investigated by objectively assessing the physical activity behaviors, physiological characteristics, and sleep characteristics of 16 experienced male fitness trainers. Relative to the data corresponding to the preconfinement period, almost all the examined parameters exhibited significant changes during the first and second months of confinement; during the home confinement period, higher values were observed for resting heart rate and sleep latency and lower values were observed for physical activity parameters, calories spent per day, respiratory rate, and deep sleep time. All parameters returned to their preconfinement levels during the postconfinement period. The aforementioned study concluded that home confinement–induced detraining negatively affected the fitness coaches’ objective assessments of their cardiorespiratory and sleep functions [13]. Compared with these findings [36], our findings indicate that physical activity levels decreased after the implementation of stringent regulations during the Level 3 alert period and did not increase again even after the alert level was downgraded to Level 2. This inconsistency between our study and the aforementioned study may be attributed to the difference in study population (i.e., older adults vs. experienced male fitness trainers, respectively). When our participants could not maintain their physical activity habits, they found it difficult to return to their previous physical activity levels.

The COVID-19 pandemic and the subsequent home confinement measures that were implemented had a profound effect on mental health [24–26]. During the COVID-19 outbreak in China, mental health burden (anxiety, depression, and sleep quality) was evaluated in 7236 individuals [26]; anxiety, depression, and poor sleep quality were prevalent in 35.1%, 20.1%, and 18.2% of the population, respectively, and higher incidences of anxiety and depression were noted in participants aged < 35 years than in those aged > 35 years. Compared with other types of professionals, an increased proportion of health-care professionals reported poor sleep [26]. A study reported that home confinement negatively affected mental well-being, moods, and feelings; the participants in that study reported poorer mental well-being during the home confinement period than during the preconfinement period; the difference was 12.89%. Furthermore, a mood and feelings survey conducted in the aforementioned study revealed a 44.9% increase in total score (e.g., a higher total score indicates more severe depressive symptoms), with 10% more participants experiencing depressive symptoms during the home confinement period than during the preconfinement period [24]. A global survey of 1681 Muslim athletes revealed that the lockdown reduced their quality of sleep and exacerbated their insomnia [9]. The respondents reported taking longer and later daytime naps and eating more late-night meals during the lockdown period than during the prelockdown period; all of these factors are associated with poor sleep quality [9]. A study involving 3323 individuals explored how COVID-19-related public health regulations affected the participants’ anxiety, well-being, and sleep quality [12]. Compared with the data of sedentary participants, active participants reported improved well-being, enhanced sleep quality, and reduced anxiety. These findings suggest the positive effects of physical activity on well-being, anxiety, and sleep quality during the period when COVID-19 restrictions were implemented [16]. Our findings are consistent with those of some studies but are different from those of some other studies. In the present study, the participants’ overall BSRS-5 score was < 5, indicating that Taiwan’s national epidemic prevention measures did not have a substantial psychological effect on older adults who regularly participated in physical activities. Notably, when the alert level was downgraded from Level 3 to Level 2, the participants reported higher levels of anxiety because the Taiwanese government opted to coexist with the virus instead of maintaining its zero-COVID policy. The older adults who resided in Yilan County reported increased anxiety when the number of confirmed cases increased. Furthermore, the participants experienced higher levels of inferiority during the Level 3 alert period than during the Level 2 alert period. This is because participants who were physically active before the lockdown were concerned about the functional decline due to inactivity during the lockdown period.

Because COVID-19 was controlled well in Taiwan by June 2021, the Taiwanese government downgraded the alert level to Level 2 on June 27, 2021. Consequently, the restrictions were loosened; however, the risk of viral transmission increased. Subsequently, COVID-19 spread rapidly to various communities; the number of people infected increased in May 2022. Nevertheless, Taiwan’s Central Epidemic Command Center did not upgrade the alert level again. However, most people in Taiwan strengthened their epidemic prevention behaviors, including those relating to physical activities. Depending on the agility of policy implementation, the balance between the normalization of activity in a community and the prevention of an epidemic may be achieved. The present study highlights the effects of COVID-19 on the physical activity behaviors and psychological distress of community-dwelling older adults and encourage Taiwan’s government to allow the resumption of physical activities, particularly for community-dwelling older adults.

Our study has some limitations. First, we sampled community-dwelling older adults who were functionally independent and engaged in physical activities; all participants resided in Yilan County, which is less densely populated and urbanized than various other parts of Taiwan. Therefore, our findings may not be generalized to populations living in urban or metropolitan areas. Older adults who reside in large cities and those who do not participate in physical activities should also be evaluated in future studies. These factors could have introduced bias, and they limit the generalization of our findings to other populations. Second, although several repeated surveys were conducted in this longitudinal study to address a previous gap pertaining to the cross-sectional design, a limitation is the fewer number of participants surveyed during the Level 2 alert period than during the Level 3 alert period. The number of confirmed cases and that of people who received vaccines caused the government’s plans to shift even when the alert level did not change. Thus, we were limited to interviewing as many individuals as we could under similar circumstances. Finally, to avoid recall bias, we did not use the BSRS-5 during the pre-COVID-19 period; hence, the baseline information on mental health might be insufficient.

## Conclusions

Our results indicated that Taiwan’s COVID-19 alert levels affected the physical activity behaviors and psychological distress of community-dwelling older adults. During the Level 3 alert period, the participants continued their physical activities but focused on solitary activities. Although the Level 3 alert was downgraded to Level 2 alert, the older adults’ physical activity levels remained low because they had to adhere to strict regulations. Regarding mental health, we noted a slight decrease in the participants’ overall BSRS-5 scores when the Level 3 alert was downgraded to Level 2 alert. However, the participants experienced considerably more anxiety and inferiority during the Level 2 alert period than during the Level 3 alert period. Time is required for older adults to regain their prior status after their physical activity behaviors and psychological distress were affected by national regulations.

## Data Availability

The data that support the findings of this study are not publicly available because their containing information that could compromise the privacy of research participants but are available from the corresponding author upon reasonable request.
